# Evaluation of Lipid Extracts from the Marine Fungi *Emericellopsis cladophorae* and *Zalerion maritima* as a Source of Anti-Inflammatory, Antioxidant and Antibacterial Compounds

**DOI:** 10.3390/md21040199

**Published:** 2023-03-23

**Authors:** Mariana Abraúl, Artur Alves, Sandra Hilário, Tânia Melo, Tiago Conde, Maria Rosário Domingues, Felisa Rey

**Affiliations:** 1 ECOMARE—Laboratory for Innovation and Sustainability of Marine Biological Resources, CESAM—Centre for Environmental and Marine Studies, Department of Chemistry, University of Aveiro, Campus Universitário de Santiago, 3810-193 Aveiro, Portugal; 2 LAQV-REQUIMTE, Mass Spectrometry Centre, Department of Chemistry, University of Aveiro, Campus Universitário de Santiago, 3810-193 Aveiro, Portugal; 3 CESAM—Centre for Environmental and Marine Studies, Department of Biology, University of Aveiro, Campus Universitário de Santiago, 3810-193 Aveiro, Portugal

**Keywords:** antibacterial, anti-inflammatory, antioxidant activity, α-linolenic acid, COX-2, fatty acids, lipids, marine fungi, PUFA

## Abstract

Marine environments occupy more than 70% of the earth’s surface, integrating very diverse habitats with specific characteristics. This heterogeneity of environments is reflected in the biochemical composition of the organisms that inhabit them. Marine organisms are a source of bioactive compounds, being increasingly studied due to their health-beneficial properties, such as antioxidant, anti-inflammatory, antibacterial, antiviral, or anticancer. In the last decades, marine fungi have stood out for their potential to produce compounds with therapeutic properties. The objective of this study was to determine the fatty acid profile of isolates from the fungi *Emericellopsis cladophorae* and *Zalerion maritima* and assess the anti-inflammatory, antioxidant, and antibacterial potential of their lipid extracts. The analysis of the fatty acid profile, using GC-MS, showed that *E. cladophorae* and *Z. maritima* possess high contents of polyunsaturated fatty acids, 50% and 34%, respectively, including the omega-3 fatty acid 18:3 *n*-3. *Emericellopsis cladophorae* and *Z. maritima* lipid extracts showed anti-inflammatory activity expressed by the capacity of their COX-2 inhibition which was 92% and 88% of inhibition at 200 µg lipid mL^−1^, respectively. *Emericellopsis cladophorae* lipid extracts showed a high percentage of inhibition of COX -2 activity even at low concentrations of lipids (54% of inhibition using 20 µg lipid mL^−1^), while a dose-dependent behaviour was observed in *Z. maritima*. The antioxidant activity assays of total lipid extracts demonstrated that the lipid extract from *E. cladophorae* did not show antioxidant activity, while *Z. maritima* gave an IC_20_ value of 116.6 ± 6.2 µg mL^−1^ equivalent to 92.1 ± 4.8 µmol Trolox g^−1^ of lipid extract in the DPPH• assay, and 101.3 ± 14.4 µg mL^−1^ equivalent to 106.6 ± 14.8 µmol Trolox g^−1^ of lipid extract in the ABTS•^+^ assay. The lipid extract of both fungal species did not show antibacterial properties at the concentrations tested. This study is the first step in the biochemical characterization of these marine organisms and demonstrates the bioactive potential of lipid extracts from marine fungi for biotechnological applications.

## 1. Introduction

The interest in the identification of new therapeutic agents has guided the exploration of natural environments throughout human history. Marine ecosystems occupy more than 70% of the earth’s surface, presenting very diverse environments and singular conditions. Marine habitats have been colonized by highly differentiated organisms, and much of them remain unexplored. However, technological advances have revealed that these habitats contain organisms that can be used as sources of diversified natural products.

Marine organisms are a source of bioactive compounds, being increasingly studied due to their beneficial properties for biotechnological applications [1,2,3,4]. From 2016 to 2020, 7547 new natural products of marine origin have been described [5]. In this search for new natural compounds, microorganisms have stood out for their potential due to their ability to be cultivated on a large scale, producing high amounts of primary and secondary metabolites at low cost and with low environmental impact [1,6].

Marine fungi are an ecologically diverse group found in nearly every marine habitat explored, such as decaying coastal wood, algae, marine animals, coral reef, sea garbage, mangrove plants, or sediments [7,8,9,10,11]. These organisms play relevant ecological roles contributing to phytoplankton population cycles, carbon pumps, and nutrient cycling [12]. Several fungal species are used for different purposes, such as food, medical or industrial applications [13,14]. Enzymes (e.g., polysaccharidases, lipases, proteases) extracted from marine fungi have been studied due to their specific activity, even under less favorable conditions (e.g., high salinity, high pressure, acid, and alkaline pH) [13]. Despite the importance of their metabolites for scientific advances, the exploitation of marine fungi to produce bioactive compounds is limited [15,16]. However, marine fungi represent the marine group with the highest number of new compounds analyzed in recent years, illustrating the progressive interest in the identification of bioactive compounds produced by these organisms [5,17]. These compounds included molecules from eight groups: terpenes, sterols, alkaloids, ethers, phenols, lactones, peptides, and others that do not fit into these groups [3,15]. Several molecules produced by marine fungi have been identified for their anti-inflammatory, antibacterial, antiviral, and anticancer properties [15,17,18]. Fungi represent cheap and easily available sources of bioactive compounds, such as lipids, enzymes, or organic acids [19,20]. These marine organisms could be used as bio-factories to produce compounds with bioactivity for biotechnology industries.

Inflammatory diseases are one of the most common pathologies nowadays, so it is necessary to find new sources of anti-inflammatory agents. A recent review of marine fungi identified 133 anti-inflammatory metabolites, polyketides, and terpenoids, the chemical classes with a high number of reported molecules [17]. However, the anti-inflammatory potential of lipids of fungal origin has been poorly studied, even though strong anti-inflammatory and anti-thrombotic properties have been found in lipid fractions of the entomopathogenic fungus *Beauveria bassiana* (Bals.-Criv.) Vuill. [21].

Inflammation and oxidative processes are closely linked to many diseases and disorders (e.g., cardiometabolic disease, obesity, depression) [22,23,24], as the inflammatory response can lead to increased oxidative stress. Then, the balance of both processes is important to maintain human health. The imbalance between the production of reactive oxygen species (ROS), either generated by endogenous (e.g., mitochondria) or exogenous (e.g., environmental factors, pollutants, radiations) sources [25], and the endogenous antioxidant compounds can lead to oxidative stress, causing damage to fatty acids, DNA and proteins as well as other cellular components [25]. ROS imbalance has been associated with several disorders in human health, such as rapid aging, cancer, cardiovascular, inflammatory, and neurodegenerative diseases [26,27]. Therefore, the search for alternative sources of natural antioxidants for the pharma, cosmetic, and food industries has been increasing in the last few years.

Natural compounds with antioxidant activity have been explored in marine organisms [28,29,30]. These compounds are interesting natural ingredients for several fields, such as food preservatives, functional ingredients to inhibit the action of ROS, preventing the oxidative damage associated with several diseases [31], or cosmetics to protect against oxidative injuries associated with UV irradiation and photoaging [32]. The antioxidant activity of methanolic extracts from marine fungi has been identified in several species, such as *Cladosporium cladosporioides* (Fresen.) G.A. de Vries and *Curvularia trifolii* (Kauffman) Boedijn from corals [33], and *Cladosporium rubrum* T. Vicente, M. Gonçalves & A. Alves and *Penicillium lusitanum* M. Gonçalves, L. Santos, B.M.V. Silva, A.C. Abreu, T.F.L. Vicente, A.C. Esteves & A. Alves from algae and seawater, respectively [19]. Moreover, some compounds, such as polysaccharides isolated from marine filamentous fungi (e.g., *Penicillium* sp.) have also been identified as antioxidant agents [34]. However, the activity of lipid extracts from marine fungi has been scarcely investigated.

The objective of the present study was to characterize the fatty acid profile of two species of marine fungi and to screen the bioactive potential of their lipid extracts as anti-inflammatory, antioxidant, and antibacterial agents. The model species for this study were the fungi *Emericellopsis cladophorae* M. Gonçalves, T. Vicente & A. Alves, and *Zalerion maritima* (Linder) Anastasiou, which have been associated with interesting properties [19,35]. *Emericellopsis cladophorae* belongs to the family *Bionectriaceae*. This species has been recognized to have antibacterial, antioxidant, and cytotoxic bioactivities [19]. A recent study with *E. cladophorae* using untargeted metabolomics and genome sequencing determined the biosynthetic potential of this fungal species to produce bioactive metabolites [36]. *Zalerion maritima* belongs to the family *Lulworthiaceae*. This species has been shown to produce extracellular enzymatic activities (e.g., amylases, cellulase, xylanase), and mycelium extracts exhibited antibacterial activity against Gram-positive bacteria [19]. However, the lipid composition of these fungal species has been overlooked.

## 2. Results

### 2.1. Fatty Acid Profiles

The lipid content in *E. cladophorae* and *Z. maritima* was 3.30 ± 2.64 and 1.60 ± 0.40 µg lipid mg^−1^ wet weight, respectively. The percentage of phospholipids in total lipid extracts was 6.16% ± 2.92 and 29.01% ± 13.31 in *E. cladophorae* and *Z. maritima*, respectively.

Fatty acid profiles of the lipid extracts of *E. cladophorae* and *Z. maritima* are summarized in Table 1. This analysis revealed that the most abundant fatty acids in *E. cladophorae* samples were 18:2 *n*-6 (45.38 ± 4.79%), 18:1 *n*-9 (18.35 ± 0.59%), 16:0 (15.97 ± 0.73%), 18:0 (13.51 ± 5.55%) and 18:3 *n*-3 (4.22 ± 0.69%) (Table 1). The fatty acids with the highest relative abundance in the *Z. maritima* samples were 16:0 (27.64 ± 2.72%), 18:0 (26.43 ± 9.34%), 18:2 *n*-6 (24.51 ± 7.32%), 18:1 *n*-9 (11.07 ± 3.20%) and 18:3 *n*-3 (7.95 ± 1.47%) (Table 1). In both fungal species, these fatty acids represented more than 97% of the total fatty acids. The odd-chain fatty acids 15:0 and 17:0 were identified in trace abundances (~0.2%) in both species.

*Emericellopsis cladophorae* presented a high relative abundance of unsaturated fatty acids (~69%), containing 49.85% ± 5.45 polyunsaturated fatty acids (PUFA) and 18.99 ± 0.58% of monounsaturated fatty acids (MUFA), while saturated fatty acids (SFA) accounted for 31.16 ± 5.10% (Table 1). *Zalerion maritima* has a high abundance of SFA (55.37 ± 11.75%), with 11.78 ± 3.40% and 32.55 ± 8.36% of MUFA and PUFA, respectively (Table 1).

### 2.2. Anti-Inflammatory Activity

**Figure 1:**
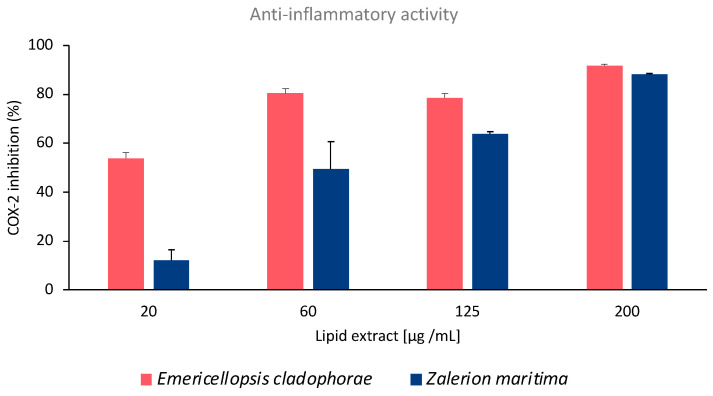
Inhibition of COX-2 activity (expressed as percentage of inhibition, %) as a function of the concentration of the lipid extracts of *Emericellopsis cladophorae* and *Zalerion maritima*. Results are averages of three assays (*n* = 3) ± standard deviation.

The anti-inflammatory potential of *E. cladophorae* and *Z. maritima* lipid extracts was evaluated by the inhibition of human cyclooxygenase-2 (COX-2) activity assay. Lipid extracts of *E. cladophorae* showed high inhibition of COX-2 activity even at low concentrations (Figure 1). The extract of this fungal species showed inhibition of COX-2 activity of 53.9 ± 2.4%, 80.3 ± 2.0%, 78.5 ± 1.9%, and 91.7 ± 0.6% with concentrations of lipid extracts of 20, 60, 125 and 200 µg mL^−1^, respectively (Figure 1). The lipid extract required to inhibit 60% (IC_60_) of COX-2 activity was 45.38 ± 10.68 µg mL^−1^. The anti-inflammatory activity of *Z. maritima* lipid extracts exhibited a dose-dependent behavior with an increment in the inhibition of COX-2 activity at increasing lipid concentrations (Figure 1). The inhibition of COX-2 activity was 12.3 ± 4.3%, 49.4 ± 11.2%, 63.9 ± 0.6%, and 88.2 ± 0.2% at concentrations of 20, 60, 125, and 200 µg lipid mL^−1^, respectively (Figure 1). In this fungal species, the IC_60_ was 124.44 ± 1.55 µg lipid mL^−1^.

### 2.3. Antioxidant Activity

The total lipid extracts of *E. cladophorae* did not show antioxidant activity. Antioxidant activity was observed in *Z. maritima* lipid extracts, requiring concentrations of lipid extracts of 116.63 ± 6.21 and 101.30 ± 14.37 µg mL^−1^ to inhibit 20% (IC_20_) of DPPH• and ABTS•^+^ radicals, respectively (Table 2). The Trolox equivalent was calculated for DPPH• and ABTS•^+^ assays, with values being 92.13 ± 4.82 and 106.58 ± 14.75 µmol Trolox g^−1^ lipid extract, respectively (Table 2).

### 2.4. Antibacterial Activity

**Figure 2:**
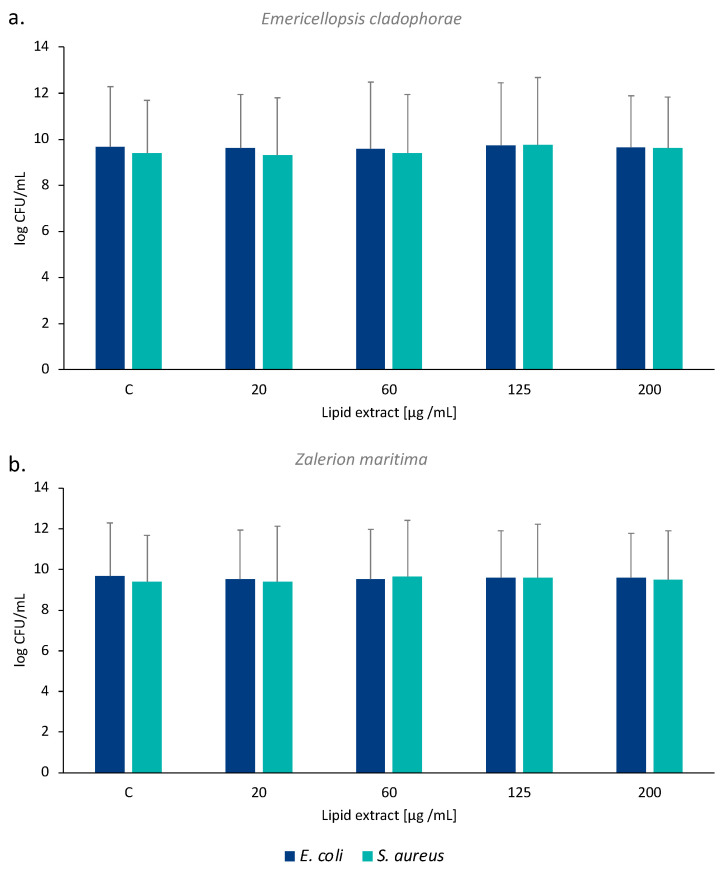
Effects of (**a**) *Emericellopsis cladophorae* and (**b**) *Zalerion maritima* lipid extracts on the growth of *Escherichia coli* and *Staphylococcus aureus*, expressed as log CFU mL^−1^. Values are averages of three assays (*n* = 3) ± standard deviation.

The total lipid extract of *E. cladophorae* and *Z. maritima* did not show bacteriostatic or bactericidal effects on both bacteria tested, *Escherichia coli* (ATCC 25922) and *Staphylococcus aureus* (ATCC 6538). The log UFC mL^−1^ values were identical between the different concentrations of lipid extract tested and the control (Figure 2).

## 3. Discussion

The lipid yield of *E. cladophorae* was higher than that of *Z. maritima*. However, the percentage of phospholipids in the lipid extracts of *Z. maritima* was higher than that in *E. cladophorae*, representing about a fourth of the total lipids in the former species. Marine fungi exhibit elevated lipid content, with a lipid: carbohydrate: protein ratio of c. 13:5:1, on average [37]. Several species of fungi have been recognized as oleaginous microorganisms due to the high lipid content, which can represent up to 86% of dry weight in some species [38]. The lipid content in fungi is influenced by biotic (e.g., species strain) and abiotic factors (e.g., nitrogen and carbon sources, C/N ratio, temperature, pH) [6,38]. Then, the yield of triacylglycerols (TAG) and the fatty acid composition are modeled by growth conditions and may vary between isolates of fungi, as it was encompassed in several strains [6]. Additionally, the lipid composition changes according to mycelia age. Young mycelia presented a higher proportion of polar lipids (e.g., glycolipids, sphingolipids, and phospholipids) and PUFA; however, an increment in neutral lipid (e.g., TAG) was observed in aged mycelia [39]. A study comparing thirteen marine fungal species isolated from the water column and sediments identified phospholipids as the main polar lipid category, with phosphatidylcholine (PC) and phosphatidylethanolamine (PE) as the most abundant phospholipid classes [40].

*Emericellopsis cladophorae* displayed a high amount of PUFA, with a relative abundance of almost 50%. However, in *Z. maritima*, the most abundant fatty acid group was SFA. Studies in marine fungi have identified the fatty acid 18:2 *n*-6 as the most abundant PUFA, with a relative abundance between 15–55% [40,41]. Similar results have been obtained in the present study. The α-linolenic acid (18:3 *n*-3, ALA) was the second most abundant PUFA in both *E. cladophorae* and *Z. maritima* species, with a relative abundance higher than that observed in other species [41]. However, the fatty acid profile of the same strain can differ according to the age of the cultures, showing that the stationary phase may provide a more reproducible fatty acid composition than younger cultures [41]. The most abundant PUFA identified in *E. cladophorae* and *Z. maritima* (i.e., 18:2 *n*-6 and 18:3 *n*-3) are important fatty acids in marine food webs since they are precursors of essential fatty acids such as arachidonic acid (20:4 *n*-6, ARA), eicosapentaenoic acid (20:5 *n*-3, EPA) and docosahexaenoic acid (22:6 *n*-3, DHA) [42]. ALA has also been noted for playing essential roles in human health, such as brain development [43], cardiovascular-protective agents [44], or skin lipid modulators improving the skin barrier [45]. Marine fungi can be an alternative source of ingredients of omega-3 fatty acids, namely the essential fatty ALA, for healthy and nutritional products.

The evaluation of COX-2 activity and expression is one of the most common assays in screenings for anti-inflammatory potential. The COX-2 enzyme is selectively induced by pro-inflammatory mediators during inflammation, such as cytokines, mitogens, carcinogens, or oncogenes. This enzyme catalyzes prostaglandin biosynthesis from 20:4 *n*-6, whose availability is dependent on phospholipase A2 (PLA2) expression and/or activity [46]. Prostaglandins are eicosanoids with important roles as pro-inflammatory signaling molecules. Thus, the inhibition of the COX-2 enzyme promotes the suppression of prostaglandins and, consequently, an anti-inflammatory effect. The lipid extracts of *E. cladophorae* showed a high (54%) inhibition of COX-2 activity with the lowest concentration tested (20 µg lipid mL^−1^) while inhibited 90% of COX-2 activity with the highest concentration tested (200 µg lipid mL^−1^). Fatty acid analysis of *E. cladophorae* and *Z. maritima* identified omega-3 fatty acids in their lipid extracts, which play a key role as regulators of the inflammatory process. These molecules compete with ARA to produce eicosanoids, suppressing the production of pro-inflammatory mediators [47], and acting as antagonist agents. The beneficial effect of ALA (18:3 *n*-3) against inflammatory-related diseases has been suggested in several studies. Exposure of macrophages to the fatty acid 18:3 *n*-3 promoted a high increase in oxylipins derived from this fatty acid, suggesting that ALA may dampen the inflammatory phenotype of M1-like macrophages [48]. Consumption of diets rich in ALA contributed to potentially beneficial alterations in the plasma oxylipin profiles by reducing oxylipins that induce inflammation [49]. ALA isolated from *Actinidia polygama* fruit downregulates the inflammatory iNOS, COX-2, and TNF- α gene expressions and modulates the anti-inflammatory response [50]. Further studies to elucidate the lipid molecules in which 18:3 *n*-3 is esterified will reveal the potential of these fungal lipid extracts as anti-inflammatory agents in therapeutic treatments.

The lipid extracts of *E. cladophorae* did not show antioxidant activity in the present study. This result is in accordance with the low antioxidant activity obtained in a previous study using methanolic mycelial extracts, which also contain lipids [19]. The differences in antioxidant activity found between the present study (no activity) and that observed in the previous study (low activity of methanolic extracts) could be related to different extract compositions. Methanolic extracts can contain other molecules, like phenolics, that could contribute to the low activity observed. The antioxidant activity of *E. cladophorae* has been related to phenolic, ortho-phenols, and flavonoid compounds [19], as observed in other filamentous fungi [51,52]. Additionally, the differences can also be associated with physiological effects related to the life stage or culture conditions [51,53], as microorganisms can produce different compounds throughout their life stages or under different growth conditions [6,38]. Other extracts from *E. cladophorae*, such as culture filtrate extracts, showed antibacterial activity against the Gram-positive bacterium *Kocuria rhizophila*, while culture medium extracts reduced between 30% and 70% the viability of Vero cells in cytotoxicity assays [19].

The results of the antioxidant scavenging activity of *Z. maritima* lipid extracts against DPPH radicals suggest a higher antioxidant activity than other strains of marine fungi analyzed previously [19]. Studies evaluating the antioxidant activity of other filamentous fungi achieved an IC_50_ in ABTS assays using concentrations of methanolic or ethanol extracts between 2 and 13 mg mL^−1^ [51,52]. However, the present study showed that lipid extracts of *Z. maritima* achieved an IC_20_ in ABTS assays with a concentration of 101 µg mL^−1^. These results suggest a higher antioxidant capacity of this marine species than that of filamentous fungi isolated from other sources. Marine organisms are recognized to have a high proportion of PUFA. These fatty acids are associated with several beneficial properties, such as antioxidant activity [54]. A study screening the antioxidant activity of lipid extracts from seven microalgae determined that higher antioxidant activity was associated with a higher concentration of PUFA [55]. Omega-3 PUFA, such as 18:3 *n*-3, act as antioxidants by regulating the antioxidant signaling pathways of cell membranes [54]. The higher relative abundance of 18:3 *n*-3 in *Z. maritima* than in *E. cladophorae* may contribute to the antioxidant activity of the former species.

Several factors have led to the development of antimicrobial resistance microbes, such as the misuse and overuse of antibiotics. The identification of new antimicrobial drugs to combat antibiotic-resistance microorganisms (e.g., *S. aureus*, *Mycobacterium tuberculosis*, *Pseudomonas aeruginosa*) is urgently needed. The lipids identified in marine organisms have been shown to be a natural source of effective agents against a wide spectrum of microorganisms [4,56]. The lipid extracts of *E. cladophorae* and *Z. maritima* did not show antibacterial activity against *E. coli* ATCC 25922 (Gram-negative) and *S. aureus* ATCC 6538 (Gram-positive) bacteria. Although *E. cladophorae* showed activity against the Gram-positive bacterium *K. rhizophila* in a previous study [19], this was based on culture medium extracts and may be associated with secondary metabolites secreted by the fungus. In fact, species of *Emericellopsis* have been shown to produce metabolites with antimicrobial properties, such as nonribosomal peptides [57].

PUFA have been recognized for their bioactivity and healthy benefits. However, the biological activities recorded in the lipid extracts of *E. cladophorae* and *Z. maritima* can be related to different lipid classes or species or by a synergistic effect of different lipids present in their lipidome. An in-depth characterization of lipid extracts using lipidomics tools would shed some light on the structure of bioactive lipids and be useful for understanding the mechanisms of action and the structure-activity relationship.

## 4. Materials and Methods

### 4.1. Reagents

Potato Dextrose Broth and Potato Dextrose Agar were purchased from Merck (Darmstadt, Germany), and sea salt from Sigma-Aldrich (St. Louis, MO, USA). Dichloromethane (CH_2_Cl_2_) and methanol (MeOH) were purchased from Fisher Scientific Ltd. (Loughborough, UK), and *n*-hexane was purchased from Carlo Erba Reagents (Cornaredo, MI, Italy). All the solvents were of high-performance liquid chromatography (HPLC) grade. Milli-Q water was used as ultrapure water (Synergysup^®^, Millipore Corporation, Billerica, MA, USA). 2,2-diphenyl-1-picrylhydrazyl radical (DPPH•) was purchased from Aldrich (Milwaukee, WI); 2,20-Azino-bis(3-ethylbenzothiazoline-6-sulfonic acid) diammonium salt (ABTS•^+^) was obtained from Fluka (Buchs, Switzerland); 6-hydroxy-2,5,7,8-tetramethylchromane-2-carboxylic acid (Trolox) was purchased from Sigma-Aldrich (St. Louis, MO, USA). The cyclooxygenase (COX-2) inhibitory screening assay was performed using a commercial kit, Cayman test kit-701080, from Cayman Chemical Company (Ann Arbor, MI, USA). All the other reagents and chemicals used were of the highest grade of purity commercially available.

### 4.2. Fungal Strains

Two fungal strains isolated from the Portuguese coast were used in this study. The *E. cladophorae* strain (MUM 19.33) was identified by Gonçalves et al. [8]. It was isolated from the green macroalga *Cladophora* sp., in the estuary of Ria de Aveiro [8]. The *Z. maritima* strain (CMG 67) was previously identified by Gonçalves et al. [58]. This strain was isolated from wood blocks of *Pinus pinaster*, which were submerged three meters deep in a marina in Ria de Aveiro for one year [58].

### 4.3. Strain Cultivation

The fungal strains were cultivated in Potato Dextrose Broth (PDB) containing 3% sea salt, which was prepared and distributed in 50 mL portions over seven 250 mL Erlenmeyer flasks, autoclaved at 120 °C for 20 min at 1 bar pressure. Three plugs (± 5 mm), taken from cultures of *E. cladophorae* and *Z. maritima* actively growing in Potato Dextrose Agar (PDA) (Merck, Darmstadt, Germany) containing 3% sea salt, were introduced into each Erlenmeyer for inoculation. Fungal cultures were incubated at 25 °C for 14 days.

After mycelium growth, the biomass was collected by gravitational filtration through filter paper and placed into 50 mL tubes. Four biological replicas (*n* = 4) of *E. cladophorae* and *Z. maritima* biomass were obtained and immediately stored at −80 °C for further analysis.

### 4.4. Lipid Extraction

The frozen mycelium was macerated with liquid nitrogen using mortar and pestle. The total lipids were extracted from the mycelium using the Bligh and Dyer method [59] with some modifications. Briefly, the biomass was mixed with 2.5 mL of methanol and 1.25 mL of dichloromethane and homogenized for 2 min, followed by sonication for 15 min. Subsequently, the mixture was incubated on ice for 60 min in an orbital shaker (Stuart, SSL2) at 100 rpm. During the incubation period, it was sonicated for 15 min after 30 min and 60 min. After this period, 1.25 mL of dichloromethane was added to the mixture, and it was homogenized for 2 min and centrifugated (Centurion Scientific, Pro-Analytical C4000R, Stoughton, UK) at 3000 rpm for 10 min. The organic phase was recovered in a new glass tube. The biomass was reextracted by adding 2.5 mL of methanol and 2.5 mL of dichloromethane to the pellet, followed by homogenization and centrifugation at 3000 rpm for 10 min. The organic phase was collected and combined with the first in the previous tube. After drying, 2.5 mL of methanol, 2.5 mL of dichloromethane, and 2.25 mL of Milli-Q water were added. Then the mixture was vortexed for 2 min and centrifuged at 3000 rpm for 10 min. The organic phase was collected, and the aqueous phase was reextracted by adding 1.8 mL of dichloromethane, homogenized for 1 min, and centrifugated at 3000 rpm for 10 min. The organic phase was collected into the respective tube and dried under a nitrogen stream. The lipid extracts were transferred to amber vials, dried, weighed, and stored at −20 °C for further analysis. The total lipid extract was quantified by gravimetry.

### 4.5. Phospholipid Quantification

The amount of phospholipid was quantified according to Bartlett and Lewis [60]. Lipid extracts were dissolved in 300 μL of dichloromethane, and 10 μL of each sample was transferred into a glass tube, in duplicate, and dried under a nitrogen stream. A volume of 125 μL of perchloric acid (70%) was added, and the samples were placed in a heating block (Stuart, SBH200D/3) at 180 °C for 60 min to promote phospholipid hydrolysis. Phosphate standards (0 to 1.5 μg mL^−1^) were prepared for the calibration curve. To all samples and standards were added 825 μL of Milli-Q water, 125 μL of ammonium molybdate (NaMoO_4_ H_2_O, 2.5%), and 125 μL of ascorbic acid (10%), followed by a 1-min vortex homogenization between additions. Both samples and standards were incubated in a water bath at 100 °C for 10 min. After cooling, 200 µL of samples and standards were transferred to a 96-well reading plate, and absorbance was measured at 797 nm on a microplate UV-Vis spectrophotometer. The conversion factor 775/31 (25) was used to estimate the phospholipid amount in the samples.

### 4.6. Fatty Acid Analysis through Gas Chromatography—Mass Spectrometry (GC-MS)

#### 4.6.1. Transesterification

Fatty acids of total lipid extracts were transmethylated to obtain the fatty acid methyl esters (FAME). Total lipid extracts were resuspended in dichloromethane, and a volume corresponding to 8 µg of phospholipids was transferred to a glass tube previously washed with *n*-hexane. The solvent was evaporated under a nitrogen stream, and 1 mL of methylated C19:0 internal standard (0.99 μg mL^−1^), prepared in *n*-hexane, and 200 µL of a methanolic solution of potassium hydroxide (2.0 M), were added to the samples. The mixture was homogenized for 2 min, and then 2 mL of a saturated aqueous sodium chloride solution (1 g mL^−1^) was added. After centrifugation at 2000 rpm for 5 min, a volume of 600 µL of the organic phase containing the FAME was collected in a new glass tube and dried under a nitrogen stream.

#### 4.6.2. Gas Chromatography—Mass Spectrometry (GC-MS)

Analysis of the FAME was performed by gas chromatography (Agilent 8860 GC System, Santa Clara, CA, USA) coupled to mass spectrometry (GC-MS) in a GC 5977B Network Mass Selective Detector system equipped with an electronic impact source that operates at 70 eV and at a temperature of 230 °C. The column used was DB-FFAP (Agilent 123-3232, 30 m × 320 µm × 0.25 µm).

Samples were resuspended in 100 µL of *n*-hexane, and a volume of 2 µL was injected in splitless mode using a G 4513 A autosampler with the injector at 220 °C and the detector at 230 °C. The temperature program started with a temperature of 58 °C for 2 min, with a linear increase of 25 °C min^−1^ until 160 °C, followed by another increase of 2 °C min^−1^ until 210 °C and, finally, an increase of 20 °C min^−1^ until 225 °C. This temperature was maintained for 15 min. The carrier gas (helium) was maintained at a constant flow rate of 1.4 mL min^−1^. The mass spectra acquisition was performed in full scan mode in the range of *m/z* 50–550.

#### 4.6.3. Identification and Integration

Fatty acids from samples of *E. cladophorae* and *Z. maritima* were identified using Agilent MassHunter Qualitative10.0 software and the NIST14L library. Retention times and mass spectra from the samples were compared with commercial standards of fatty acid methyl esters (Supelco 37, Component FAME Mix, ref. 47885-U, Sigma-Aldrich, Darmstadt, Germany). The amounts of fatty acids were calculated as relative abundance (%) using the area of each peak obtained from integration with the Agilent MassHunter Qualitative10.0 software and considering the sum of all relative areas of the identified fatty acids.

### 4.7. Determination of Anti-Inflammatory Activity of Lipid Extracts

The anti-inflammatory potential of fungal lipid extracts was assessed using a commercial human cyclooxygenase (COX-2) inhibitor screening assay kit—Cayman test kit-701080 (Cayman Chemical Company, Ann Arbor, MI, USA). This assay measures the amount of prostaglandin F2α generated from arachidonic acid (20:4 *n*-6, ARA) in the cyclooxygenase reaction. This assay was carried out according to the instructions provided by the manufacturer. Lipid extracts of *E. cladophorae* and *Z. maritima* were dissolved in 10 µL of DMSO to obtain the final reaction concentrations of 20, 60, 125, and 200 μg of lipid mL^−1^. Positive and negative controls were provided by the assay kit protocol. The positive control used inactivated COX-2 enzyme, and the negative control used the enzyme with 100% initial activity without any inhibitor. The assay was performed in three replicates (n = 3) of lipid extracts by fungal species. Interferences were considered by subtracting COX-2 inhibition from the blank assays. The results were expressed as a percentage of inhibited COX-2 activity. The prostanoid produced was quantified by spectrophotometry (415 nm, Multiskan GO 1.00.38, Thermo Scientific, Hudson, NH, USA) and processed with the software SkanIT version 3.2 (Thermo Scientific, Waltham, MA, USA).

### 4.8. Determination of Antioxidant Activity of Lipid Extracts

#### 4.8.1. DPPH Radical Scavenging Activity Assay

The antioxidant scavenging activity against the 2,2-diphenyl-1-picrylhydrazyl radical (DPPH•) was evaluated using the Magalhães et al. method [61] with modifications [55,62]. An ethanolic solution of DPPH• (250.0 μM) was prepared. This concentration presented an absorbance of ~0.9 measured at 517 nm using a UV-Vis spectrophotometer controlled by the SkanIT software version 3.2. The stock solution was used to prepare the standard solutions (37.5, 50.0, 75.0, 100.0, and 112.50 μM in ethanol). The radical stability was evaluated after the addition of 150 µL of ethanol and 150 µL of DPPH• diluted solution in a 96-well microplate which was incubated for 120 min at room temperature, with absorbance measured at 517 nm every 5 min. The antioxidant activity of the samples was tested using three different concentrations of lipid extracts for each fungal species (20, 60, and 125 μg mL^−1^ in ethanol). The Trolox standards (5.0, 12.5, 25.0, and 37.5 μM in ethanol) were prepared using a Trolox stock solution of 1000 μM. The antioxidant scavenging potential was evaluated using a volume of 150 µL of lipid extracts at different concentrations and 150 µL of Trolox standard solutions, which were placed in the 96-well microplate and followed by the addition of 150 µL of DPPH• diluted solution. To assess the stability of the DPPH• radical, we evaluated that the absorbance variation of this solution was not greater than 10% by placing 150 µL of the DPPH• solution and 150 µL of ethanol. During the incubation with the extracts and Trolox standards, blanks were performed by replacing the DPPH• solution with 150 µL of ethanol. Radical reduction was monitored by measuring the decrease in absorbance during the reaction, quantifying the radical scavenging, accompanied by a color change. The absorbance was read every 5 min for 120 min on the UV-Vis plate spectrophotometer. All measurements were performed in triplicate.

The antioxidant activity of the lipid extracts was determined as the percentage of DPPH radical inhibition according to Equation (1):

(1) $$\mathrm{Inhibition}\;\left(\%\right)=\left[\frac{\mathrm{Abs}\;\mathrm{DPPH}-\left(\mathrm{Abs}\;\mathrm{Samples}\;-\;\mathrm{Abs}\;\mathrm{Control}\right)}{\mathrm{Abs}\;\mathrm{DPPH}\;}\;\right]\times 100$$

The concentration of the sample capable of reducing 20% of the DPPH• radical after 120 min (IC_20_) was calculated by linear regression using samples concentrations and the inhibition curve percentage. Activity is expressed as Trolox equivalents (TE, µmol Trolox g^–1^ of lipid extract), according to Equation (2):

(2) $$\mathrm{TE}\;\left({\mu \mathrm{mol}\;g}^{-1}\right)=\left[\frac{{\mathrm{IC}}_{20}\;\mathrm{Trolox}\;\left({\mu \mathrm{mol}\;L}^{-1}\right)}{{\mathrm{IC}}_{20}\;\mathrm{Samples}\;\left({\mu g\;\mathrm{mL}}^{-1}\right)}\;\right]\times 1000$$

#### 4.8.2. ABTS Radical Scavenging Activity Assay

In the 2,2’-azino-bis-3-ethylbenzothiazoline-6-sulfonic acid radical cation (ABTS•^+^) assay, an ethanolic solution of ABTS•^+^ of 7000 μM initial concentration was prepared, which was used to prepare the standard solutions (25.0, 50.0, 75.0 and 100.0 μM in ethanol). A calibration curve was constructed to calculate the ABTS•^+^ concentration corresponding to the absorbance of ~0.9, which was chosen for the samples assay. Standards were prepared by adding 150 μL of ethanol and then 150 μL of ABTS•^+^ solution to the wells, and the absorbance was read at 734 nm after 2 min on a UV-Vis plate spectrophotometer [63,64].

Three lipid extract concentrations were prepared for each fungal species (20, 60, and 125 μg mL^−1^ in ethanol). The Trolox standards (5.0, 12.5, 25.0, and 37.5 μM in ethanol) were prepared in the same way as described above. The antioxidant potential of the extracts (150 µL) and the Trolox standards activity (150 µL) were evaluated after they were placed with 150 µL of the ABTS•^+^ radical. Since the lipid extracts can absorb in the same wavelength, blanks of each extract concentration were prepared along with 150 µL of ethanol. To assess the stability of the ABTS•^+^ radical and absorbance variation, controls were performed to assess the ABTS•^+^ decay, as was done for the DPPH• assay. Radical reduction was monitored by measuring the decrease in absorbance during the reaction, quantifying the radical scavenging accompanied by a color change. The absorbance was read every 5 min for 120 min on the UV-Vis plate spectrophotometer. All measurements were performed in triplicate.

The antioxidant activity of the samples was determined as the percentage of ABTS radical inhibition according to Equation (3):

(3) $$\mathrm{Inhibition}\;\left(\%\right)=\left[\frac{\mathrm{Abs}\;\mathrm{ABTS}\;-\left(\mathrm{Abs}\;\mathrm{Samples}\;-\;\mathrm{Abs}\;\mathrm{Control}\right)}{\mathrm{Abs}\;\mathrm{ABTS}}\;\right]\times 100$$

The concentration of sample capable of reducing 20% of the ABTS•^+^ radical after 120 min (IC_20_) was calculated by linear regression using samples concentrations and the inhibition curve percentage. Activity was expressed as Trolox equivalents (TE, µmol Trolox g^–1^ of lipid extract), according to Equation (4):

(4) $$\mathrm{TE}\;\left({\mu \mathrm{mol}\;g}^{-1}\right)=\left[\frac{{\mathrm{IC}}_{20}\;\mathrm{Trolox}\;\left({\mu \mathrm{mol}\;L}^{-1}\right)}{{\mathrm{IC}}_{20}\;\mathrm{Samples}\;\left({\mu g\;\mathrm{mL}}^{-1}\right)}\;\right]\times 1000$$

### 4.9. Determination of Antibacterial Activity of Lipid Extracts

The antibacterial activity of lipid extracts was tested against two bacterial species, a Gram-positive and a Gram-negative, specifically *Staphylococcus aureus* ATCC 6538 and *Escherichia coli* ATCC 25922, respectively. The inoculum for the antibacterial tests was obtained by growing the bacteria in tryptic soy broth (TSB) at 37 °C and 150 rpm overnight (approx. 18 h). Afterward, the culture was diluted in 0.9% NaCl solution and adjusted to a concentration of 10^8^ colony-forming units (CFUs) per mL. The assays were performed in 96-well plates and in triplicate. Lipid extracts in DMSO were mixed with Mueller Hinton Broth and distributed 150 µL per well to obtain the final concentrations of 20, 60, 125, and 200 µg mL^−1^. Each well was then inoculated with 50 µL of the bacterial suspension. Two controls were included, namely, a bacterial inoculum only and a control containing the bacterial inoculum and the same amount of DMSO added to the bacterial culture with lipid extracts. The bacterial suspensions were incubated at 37 °C and 120 rpm for 24 h. After incubation, bacterial cultures were serially diluted in 0.9% NaCl and plated in Mueller Hinton agar using the drop plate method. After incubation at 37 °C for 18 h, viable counts were determined as log CFU mL^−1^ and compared with the control with DMSO. Extracts were considered to have bacteriostatic or bactericidal effects if a decrease of <3-log and ≥3-log in CFU mL^−1^, respectively, was observed.

## 5. Conclusions

The fatty acid analysis allowed the identification of a high proportion of PUFA in the composition of both fungal species (50% in *E. cladophorae*, 33% in *Z. maritima*), including interesting fatty acids such as α-linolenic acid (18:3 *n*-3). Lipid extracts of *E. cladophorae* and *Z. maritima* demonstrated a high potential as anti-inflammatory agents. *Emericellopsis cladophorae* showed a high percentage of inhibited COX-2 activity even at low concentrations of lipids (20 µg lipid mL^−1^ achieved 54% of COX-2 inhibition activity), while *Z. maritima* lipid extracts displayed a dose-dependent behavior in the inhibition of COX-2 activity. Lipid extracts of *Z. maritima* demonstrated antioxidant scavenging activity against DPPH• and ABTS•^+^ with concentrations of 116.6 ± 6.2 and 101.3 ± 14.4 µg mL^−1^, respectively to achieve an IC_20_ in both antioxidant assays. The lipid concentrations of *E. cladophorae* and *Z. maritima* used in the antibacterial assay did not show antibacterial properties against *E. coli* and *S. aureus*.

Fungi can act as cheap bio-factories of bioactive molecules, which could be cultivated on a large scale. The high proportion of PUFA in the marine fungi *E. cladophorae* and *Z. maritima* could be explored to produce food-grade lipids for food, fed, and nutraceutical ingredients. Additionally, lipid extracts of these fungal species have potential clinical applications, such as in the treatment of inflammation, wound healing, and skin diseases. This study contributes to the bioprospection of marine fungi as promising sources of natural compounds with interesting properties, which can be relevant in biotechnological applications.

## Figures and Tables

**Table 1:** Relative abundance (%) of fatty acids identified in lipid extracts of *Emericellopsis cladophorae* and *Zalerion maritima*. Values represent mean ± standard deviation (*n* = 4).

Fatty Acid	*Emericellopsis cladophorae*	*Zalerion maritima*
14:0	0.16 ± 0.01	0.43 ± 0.06
15:0	0.08 ± 0.01	0.09 ± 0.01
16:0	15.97 ± 0.73	27.64 ± 2.72
16:1	0.07 ± 0.03	—
16:1 *n*-7	0.40 ± 0.04	0.19 ± 0.09
16:2 *n*-4	0.08 ± 0.02	—
17:0	0.07 ± 0.01	0.12 ± 0.02
18:0	13.51 ± 5.55	26.43 ± 9.34
18:1 *n*-9	18.35 ± 0.59	11.07 ± 3.20
18:1	0.17 ± 0.02	0.35 ± 0.10
18:2 *n*-6	45.38 ± 4.79	24.51 ± 7.32
18:3 *n*-3	4.22 ± 0.69	7.95 ± 1.47
20:0	0.29 ± 0.11	0.33 ± 0.05
20-methyl-heneicosanoate (iso)	—	0.30 ± 0.09
20:1	—	0.17 ± 0.10
20:2 *n*-6	0.17 ± 0.04	0.12 ± 0.07
22:0	0.61 ± 0.04	—
24:0	0.48 ± 0.07	0.33 ± 0.10
SFA	31.16 ± 5.10	55.37 ± 11.75
MUFA	18.99 ± 0.58	11.78 ± 3.40
PUFA	49.85 ± 5.45	32.55 ± 8.36

Abbreviations: SFA, saturated fatty acids; MUFA, monounsaturated fatty acids; PUFA, polyunsaturated fatty acids.

**Table 2:** Inhibition concentration (IC) of lipid extracts (μg mL^−1^) providing 20% of inhibition (IC_20_) after 120 min of DPPH• and ABTS•^+^ radical scavenging activity, and the corresponding Trolox equivalent units (TE) (μmol of Trolox g^−1^ lipid) in samples from *Zalerion maritima*. Results are averages of three assays (*n* = 3) ± standard deviation.

	IC_20_ µg mL^−1^	TE µmol Trolox g^−1^ Lipid Extract
DPPH•	116.63 ± 6.21	92.13 ± 4.82
ABTS•^+^	101.30 ± 14.37	106.58 ± 14.75

## Data Availability

Data will be made available on request.
